# Summer Beverages

**Published:** 1907-06

**Authors:** 


### Summer Beverages.

WITH the approach of warm weather the inhabitants of a large city are brought to face the question of proper drinks with which to meet the unusual demand made upon the excretory functions of the body. The exhalations by the skin are more than doubled during the summer season and this excess must be met by an intake of fluid at once adequate and uninjurious.

The average householder naturally will secure an appropriate supply of "soft drinks" with which to quench the thirst of inmates and visitors. It used to be the fashion to serve lager beer when occasion required, but since it is believed that this product of the brewery contributes to the causation of certain kidney affections, this practice has been done away with to a great extent. The introduction of ginger ale, too, has served in a measure to push beer to one side. The manufacture of ginger ale in this country has increased of late to an enormous extent, and this has consequently cheapened the price of the product to a corresponding degree. It must not be forgotten, however, that the quality of the article has suffered as a result.

We advise our readers to shun the product of an unknown manufacturer of ginger ale, because there is no assurance that the quality from such sources is what it should be. If, however, the ginger ale manufactured by the Hudor Company, of Buffalo, is selected there need be no question about the high quality of the beverage. We have taken some pains to inform ourselves on this subject and believe the formula and method of manufacture of ginger ale by the Hudor Company, are on the very highest plane. It is difficult to see how these essentials could be improved upon with the present knowledge relating to the manufacture of ginger ale.

The Hudor Company also manufactures a "Brewed Ginger Beer" which is most attractive to the palate as well as free from any objectionable elements. In a very hot day this beverage appeals to many in preference to ginger ale. At all events, it is a palatable, harmless, and refreshing drink that may be taken at any and all times with confidence in its purity and high quality. The prices, too, of these refreshing beverages, ginger ale and brewed ginger beer, are quite within the reach of the modest pocketbook.

In the issue of this JOURNAL for May we discussed the question of distilled water as a substitute for all other drinking waters in the present state of the Buffalo water supply. We regard the ozonised distilled water, manufactured by the Hudor Company, as the most palatable and best drinking water for sick or well persons within the reach of the inhabitants of Buffalo.

---

THE new medical bill regulating the practice of medicine in the state of New York was signed by Governor Hughes May 13, 1907, and took effect immediately thereafter. It creates a new definition of the practice of medicine, substitutes one board of medical examiners of nine members for the three boards of seven members each, representing the several state medical societies, and recognises osteopathy as a system of treatment. The definition of the practice of medicine in the law reads as follows:

A person practises medicine within the meaning of this act, except as hereinafter stated, who holds himself out as being able to diagnose, treat, operate or prescribe for any human disease, pain, injury, deformity or physical condition, and who shall either offer or undertake, by any means or method, to diagnose, treat, operate or prescribe for any human disease, pain, injury, deformity or physical condition.

A special meeting of the Regents of the University of the State of New York is called to assemble at the capitol at Albany, May 29, 1907, for the purpose of making appointments to fill the various positions created by the new law.

---

THE next meeting of the Board of Medical Examiners for the state of Texas (regular) for examination will be held at Austin, June 25, 26 and 27, 1907. This examination will be held in accordance with the old medical law of Texas and will be the last meeting of this board for examination, as the new medical law of Texas, *the one board bill,* becomes effective July 13, and under the provisions of this law applicants will only be permitted to appear for examination who are graduates from medical colleges of not less than four terms of five months each.

---

THERE are now forty thousand automobiles registered in New York state says the *Tribune*. The time is ripe for a lightning calculator to give us statistics regarding the daily number of sudden jumps by pedestrians, swear words by chaffeurs, jokes by automobile humorists and shattered bank accounts that this figure implies.

---

BEDOUINS in Africa (*The Tribune*) have a rough and ready method of attempting to cure the fever caused by the wounds they have inflicted on those they have captured for sale as slaves. Ice baths being impossible, the patients are buried up to their necks, in the hope that the cool sand will allay the fever. They remain so buried for several days, until they have been killed or cured. Eighty per cent. succumb to the treatment.

---

A PEACE envoy who does not dare to set foot in the very land which most needs the preaching of peace is certainly handicapped in his mission.—*The Tribune.*

---

THE finding of a lost manuscript of an epic poem by Ibsen almost puts the Norwegian into the company of the classic authors.—*The Tribune.*

